# Design and implementation of three-phases energy storage system using DSP F28379D for laboratory research

**DOI:** 10.1016/j.ohx.2025.e00675

**Published:** 2025-07-05

**Authors:** Hoai Phong Nguyen, Thuan Thanh Nguyen, Minh Phuong Le, Minh Tan Tran, Cong Duy Pham

**Affiliations:** a Faculty of Electrical Engineering Technology, Industrial University of Ho Chi Minh City, Ho Chi Minh City, Viet Nam; b Department of Power Delivery, Faculty of Electronics and Electrical Engineering, Ho Chi Minh City University of Technology (HCMUT), VNU-HCM, Ho Chi Minh City, Viet Nam

**Keywords:** Three-phases energy storage system, Bidirectional 3-phase 6-switch DC/AC converter, Bidirectional DC/DC buck-boost converter, DSP F283779D

## Abstract

This paper presents the hardware design for a three-phases energy storage system connected to the grid through a safe isolation transformer, suitable for use in university laboratory experiments. The power hardware configuration includes a bidirectional DC/DC buck-boost converter and a bidirectional 3-phase 6-switch DC/AC converter. Additionally, the control board uses the Texas Instruments DSP F28379D with a charging-discharging control program written in C programming language and compiled with Code Composer Studio (CCS v12). The current and voltage sensing circuits use Hall-effect sensors to isolate the power circuit from the control circuit. A unique aspect of this research is the modular design, allowing for quick and easy upgrades and changes to the configuration and power capacity, facilitating the testing of control algorithms for the storage system. Experiments were conducted on a 3-phase 380(V) power grid through an isolation transformer and a simulated battery bank powered by the APS1000 amplifier, with a 100(V) output voltage controlled in charging mode from the grid and discharging mode to the grid at a controlled power of 230(W). The results show that the hardware model can be used effectively in laboratory settings to serve educational needs.

## Graphical abstract

**Figure:**
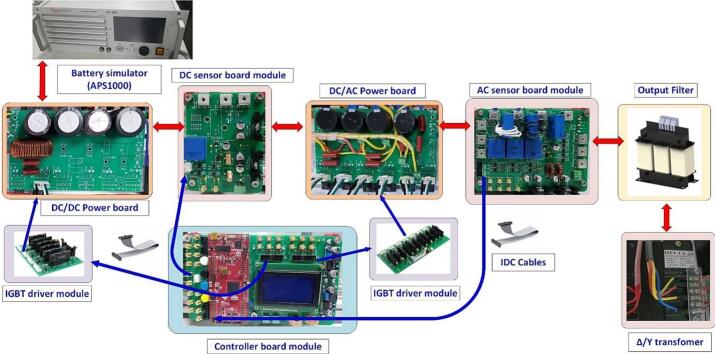

## 1 Specifications table

**Table:** 

Hardware name	Battery energy storage system
Subject area	Electrical power system Electrical and Electronic Engineering
Hardware type	Electrical engineering and computer science Electrical engineering
Closest commercial analog	The closest commercial equivalent would be compact three-phase bidirectional power inverters. The proposed design offers an open and modular architecture for custom designs, allowing the analysis of various inverter configurations and control algorithms.
Open-source license	GNU General Public License (GNU GPL v3)
Cost of hardware	Approximate total cost of hardware: 802.5$
Source file repository	https://doi.org/10.17605/OSF.IO/MZSN3

## 2 Hardware in context

The energy storage system used in the power grid with the integration of renewable energy helps to actively regulate power and store energy [1]. This device enables the storage of surplus energy, such as solar power during midday, as well as load shifting by controlling the charging and discharging of the battery in an appropriate manner [2]. There are many similar products on the market with small power ratings ranging from a few hundred watts to hundreds of kilowatts. However, most designs are integrated into a single board, which makes it difficult to modify the design. Specifically, any changes require redesigning all the components, which is time-consuming and labor-intensive.

**Fig. 1:**
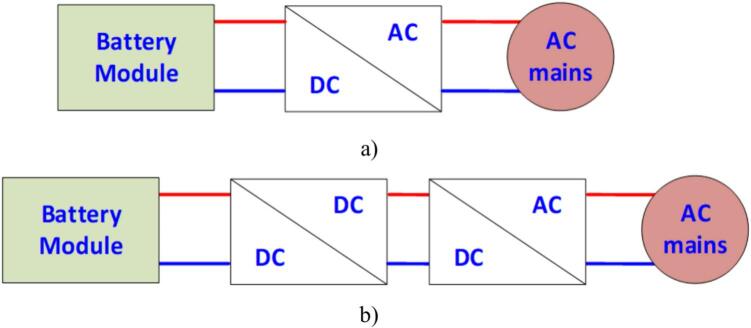
Hardware configurations of the common energy storage systems. a) The conventional Battery Energy Storage System (BESS). b) Two-stage configuration BESS.

A storage system with bidirectional power conversion capability. Depending on the application, the hardware design can be chosen in either a single-stage or two-stage configuration. Fig. 1(a) illustrates the traditional hardware configuration of the storage system. While the traditional configuration is simple, it does not function effectively with battery inputs that have very low voltage and a small operating voltage range [3]. Currently, many commercial products with a wide input voltage range are designed in a two-stage configuration as shown in Fig. 1(b). The charging direction to the battery corresponds to the power flow from the grid through the AC/DC rectifier circuit, entering the battery via the DC/DC converter for charging control. Similarly, the discharging direction to the grid involves the power flow from the battery through the DC/DC converter for discharging control and the DC/AC inverter, which synchronizes the power to the grid [4]. The control circuit reads the current and voltage signals from the system, performs the necessary algorithm calculations, and outputs PWM pulses to control the power circuits. In this system, the power control mode is set to execute a specific charging/discharging process in order to regulate the power flow when connecting the storage system to the grid.

This paper proposes the design of a modular energy storage system aimed at rapid experimentation for testing control algorithms as well as for laboratory practice. In this design, the power components, sensors, and control circuits are separated and interconnected through dedicated cables. This allows changes in power and power circuit configurations without the need to redesign the control circuit. Furthermore, the sensor circuits are carefully designed and can be configured to adjust measurement ranges by changing the feedback resistances, without altering the entire circuit. Industrial-grade heat sinks are selected, facilitating easy installation of power components via busbars. With this solution, multiple configurations and power ranges can be quickly implemented, reducing hardware development time and enabling the efficient development of control algorithms for experiments with the storage system in general.[5].

## 3 Hardware description

**Fig. 2:**
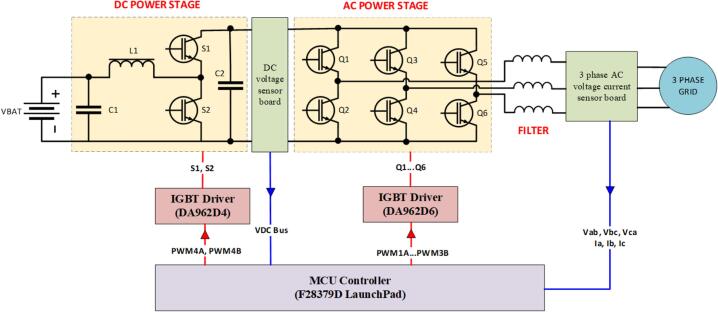
The block diagram of the proposed 3-phase energy storage system.

In this paper, a design for the energy storage system is proposed in the form of separate modules that can be connected together. This approach allows for quick assembly and modification of the hardware design for various experiments. Fig. 2 illustrates the block diagram of the hardware for a 3-phase energy storage system, with the main functions of the component circuits as follows:

(i) Bidirectional DC/DC Converter: This consists of a buck-boost circuit that converts power in both directions. The boost direction steps up the voltage from the battery for the discharging process, while the buck direction steps down the voltage from the DC bus for the charging process.

(ii) Bidirectional DC/AC Converter: This consists of a 3-phase, 6-switch circuit that converts power in both directions. The rectification direction converts the AC voltage from the 3-phase grid to DC voltage, which is then supplied to the DC circuit for battery charging. The inversion direction converts the DC voltage to AC voltage, enabling the process of feeding power from the battery back into the grid.

(iii) AC Sensing Circuit: Measures the voltage and current of the 3-phase AC grid with isolation, converting these into a readable voltage signal (0–3 V) that can be processed by the control circuit via a 14-pin header.

(iv) DC Sensing Circuit: Measures the DC voltage and current with isolation via the Hall effect on the DC busbar, converting these into a readable voltage signal (0–3 V) that can be processed by the control circuit via a 3-pin header.

(v) Driver Circuit: Receives PWM control signals and performs the switching of power components, as well as protecting the power components in case of a short circuit. In this study, the driver circuit is purchased as a pre-made module designed for IGBT switching.

(vi) Control Circuit: Designed based on the DSP F28379D LaunchPad, the control circuit includes 2 x 14-pin headers and 1 x 3-pin header, capable of reading up to 17 ADC signals with 12-bit resolution. Additionally, it integrates 2 20-pin headers that can independently generate 12 isolated PWM signals and supports display and control via an LCD screen and 4 push buttons.

**Fig. 3:**
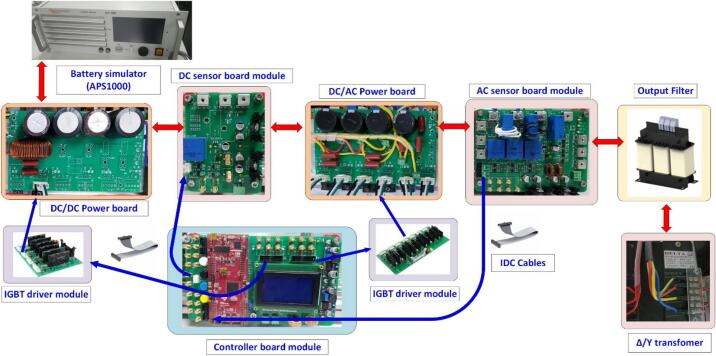
The block diagram of the experimental setup for the 3-phase energy storage system.

In the experiment, the battery is simulated using an APS1000 amplifier (4-quadrant amplifiers), with the test battery voltage set to 100 V. The DC bus voltage is controlled and stabilized at 150 V. The output of the storage system is connected to the grid safely via an isolation transformer at 50VAC. The power board is mounted on SEMIPACK heat sinks, with signal connections made through IDC cables. Fig. 3 illustrates the block diagram of the storage system setup connected to the grid through an isolation transformer in the experiment.

### 3.1 DC/DC bidirectional converter

**Fig. 4:**
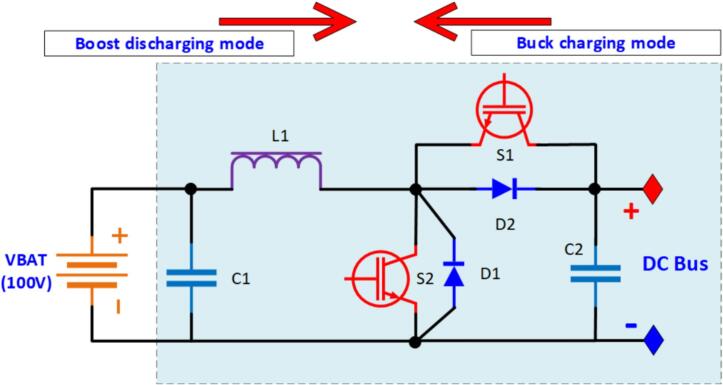
Schematic diagram of the bidirectional DC/DC power conversion circuit.

The configuration of the bidirectional DC/DC power conversion circuit includes both isolated and non-isolated types. In electric vehicle applications, bidirectional DC/DC converters are typically designed with isolation for safety reasons, whereas most other applications use non-isolated circuits. Fig. 4 presents the schematic diagram of the non-isolated bidirectional buck-boost DC/DC converter designed in this paper.

In the battery discharge mode, switch S1 is off, and the circuit operates in boost mode, where the output voltage V_DC BUS_ depends on the battery voltage V_BAT_ and the control signal of switch S2, as described by Equation (1). Conversely, in the battery charging mode, switch S2 is off, and the circuit operates in buck mode, where V_DC_BUS_ depends on the battery voltage and the control signal of switch S1, as described by Equation (2) [6].

(1) $$\mathrm{Buckchargingmode}:\left\{\begin{array}{c} D_{S1}=0\\ D_{S2}=\frac{V_{\mathrm{BAT}}}{V_{\mathrm{DCBUS}}} \end{array}\right)$$

(2) $$\mathrm{Boost}\;discharging\;mode:\;\;\left\{\begin{array}{c} D_{S2}=0\\ \frac{1}{1-D_{S1}}=\frac{V_{\mathrm{DC}\;BUS}}{V_{\mathrm{BAT}}} \end{array}\right)$$

Where, $D_{S1}$ and $D_{S2}$ are the duty cycle.

### 3.2 DC/AC bidirectional converter

**Fig. 5:**
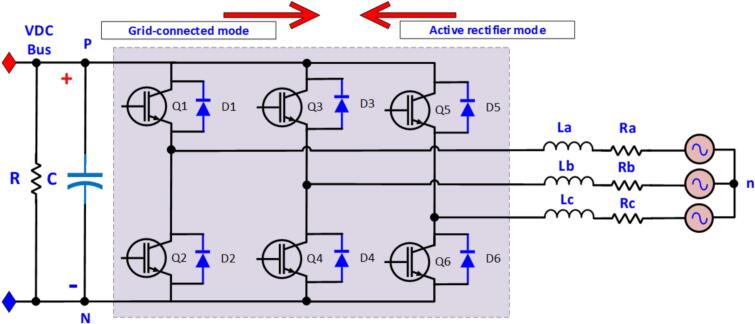
Schematic diagram of the bidirectional DC/AC power conversion circuit.

The schematic diagram of the bidirectional DC/AC power conversion circuit is shown in Fig. 5, featuring three IGBT branches. The circuit operates in grid-connected mode as a 3-phase, 6-switch inverter and in rectifier mode as a boost converter. Specifically, in rectifier mode, the voltage on the DC bus is higher than the uncontrolled 3-phase rectified voltage. In this mode, power flows from the AC grid to the DC bus voltage.

Equation (3) describes the relationship between phase current and phase voltage when the circuit operates in rectifier mode [7]. Here, $L_{a},L_{b},L_{c}\left(H\right)$ are the inductances, and $R_{a},R_{b},R_{c}(\Omega$) are the resistances of the AC-side filter inductors. The corresponding phase currents and voltages are $I_{\mathrm{la}},I_{\mathrm{lb}},I_{\mathrm{lc}}\left(A\right)$ and $U_{\mathrm{an}},U_{\mathrm{bn}},U_{\mathrm{cn}}\left(V\right)$, with *d_a_, d_b_, d_c_* (%) representing the pulse widths corresponding to switches Q1, Q2, Q3, respectively.

In grid-connected mode, power flows from the DC bus and is injected into the 3-phase grid [8]. Equation (4) describes the current and voltage response in grid-tied operation.

(3) $$\left\{\begin{array}{c} L_{a}\frac{{\mathrm{di}}_{\mathrm{La}}}{\mathrm{dt}}=-R_{a}∙{\mathrm{di}}_{\mathrm{La}}-\left(V_{\mathrm{DCBUS}}∙d_{a}+V_{\mathrm{nN}}\right)+V_{\mathrm{an}}\\ L_{b}\frac{{\mathrm{di}}_{\mathrm{Lb}}}{\mathrm{dt}}=-R_{b}∙{\mathrm{di}}_{\mathrm{Lb}}-\left(V_{\mathrm{DCBUS}}∙d_{b}+V_{\mathrm{nN}}\right)+V_{\mathrm{bn}}\\ L_{a}\frac{{\mathrm{di}}_{\mathrm{La}}}{\mathrm{dt}}=-R_{a}∙{\mathrm{di}}_{\mathrm{La}}-\left(V_{\mathrm{DCBUS}}∙d_{a}+V_{\mathrm{nN}}\right)+V_{\mathrm{cn}}\\ C\frac{{\mathrm{dV}}_{\mathrm{DC}}}{\mathrm{dt}}=d_{a}∙i_{\mathrm{La}}+d_{b}∙i_{\mathrm{Lb}}+d_{c}∙i_{\mathrm{Lc}}-\frac{V_{\mathrm{DCBUS}}}{R}\\ V_{\mathrm{nN}}=-\frac{V_{d}}{3}\left(d_{a}+d_{b}+d_{c}\right) \end{array}\right)$$

(4) $$\left\{\begin{array}{c} L_{a}\frac{{\mathrm{di}}_{\mathrm{La}}}{\mathrm{dt}}=-R_{a}∙{\mathrm{di}}_{\mathrm{La}}+\left(d_{a}+d_{n}\right)V_{\mathrm{DCBUS}}-V_{\mathrm{an}}\\ L_{b}\frac{{\mathrm{di}}_{\mathrm{Lb}}}{\mathrm{dt}}=-R_{b}∙{\mathrm{di}}_{\mathrm{Lb}}+\left(d_{b}+d_{n}\right)V_{\mathrm{DCBUS}}-V_{\mathrm{bn}}\\ L_{c}\frac{{\mathrm{di}}_{\mathrm{Lc}}}{\mathrm{dt}}=-R_{c}∙{\mathrm{di}}_{\mathrm{Lc}}+\left(d_{c}+d_{n}\right)V_{\mathrm{DCBUS}}-V_{\mathrm{cn}}\\ d_{n}=\frac{d_{a}+d_{b}+d_{c}}{3} \end{array}\right)$$

### 3.3 Power bidirectional converter board

**Fig. 6:**
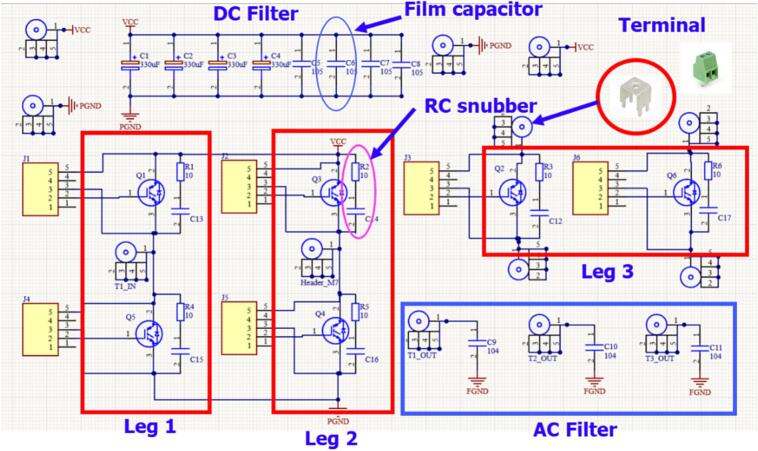
Schematic diagram of the power board.

We designed a power circuit consisting of three independent half-bridge branches to meet the requirements of a three-phase six-switch bidirectional converter. One branch is designed to be open, allowing flexible configuration with additional hardware, such as reconfiguring the power circuit into a single-phase DC/AC HERIC topology. Additionally, the circuit can be reconfigured as a DC/DC buck or boost converter. Fig. 6 illustrates the schematic diagram of the power circuit. The DC link filter capacitor includes an additional 1(uF) film capacitor to filter high-frequency switching noise. Furthermore, an RC snubber circuit is added to reduce voltage oscillation noise caused by high-frequency switching.

**Fig. 7:**
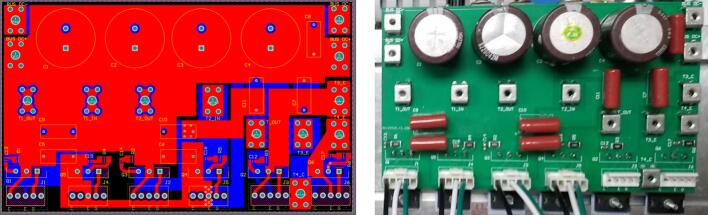
PCB layout and 3D schematic of the power board.

The printed circuit board (PCB) design emphasizes placing the high-frequency filter film capacitor as close as possible to the IGBT switch, combined with copper plating on the DC link to reduce voltage spikes caused by parasitic inductance. The gate driver header is positioned very close to the IGBT to minimize the impact of parasitic inductance on switching pulses and the overcurrent saturation voltage monitoring signal. Finally, the terminals are arranged to facilitate connections to the filter inductor and external voltage source. The PCB layout of the power converter circuit is shown in Fig. 7.

**Fig. 8:**
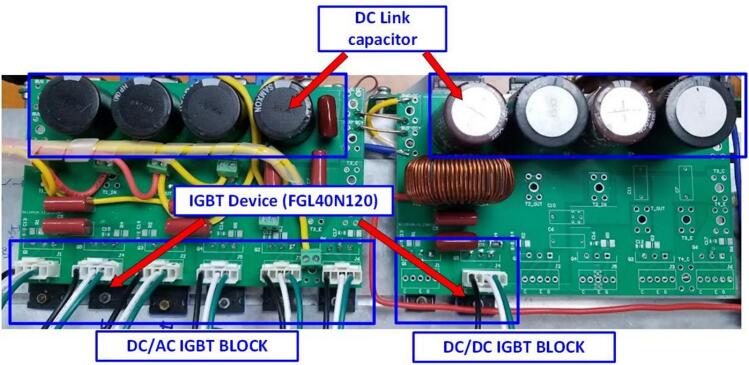
Installation of the power board onto the SEMIPACK heat sink.

In this work, the FGL40N120 IGBT device was used for both the bidirectional DC/DC converter and the bidirectional DC/AC converter. Fig. 8 illustrates the installation of the power board on the heat sink.

### 3.4 Controller board

The control circuit is built on the Texas Instruments DSP F28379D, a controller widely used in power control algorithm research. In this study, the control board is designed based on the LaunchPad F28379D, featuring the following design characteristics:

1) ADC Input: The control board can read 16 ADC input channels with 12-bit resolution via two 14-pin IDC connectors. In this proposed energy storage system, the measurement circuit is designed with six channels to monitor the three-phase grid voltage (Vab, Vbc, Vca) and the three-phase grid current (Ia, Ibc, Ic) for rectification and grid-tied operation. Additionally, two measurement channels are designed to monitor the DC bus voltage.
2) PWM Output: The control board can generate 12 optically isolated PWM output channels with 12-bit resolution via a 20-pin IDC connector. In the designed energy storage system, six PWM signals are used to control the DC/AC converter, while two PWM signals are used to control the DC/DC converter.
3) Peripherals: Supports connectivity for a 128x64 LCD, four push buttons, and an EEPROM memory IC (AT24C512BW).

**Fig. 9:**
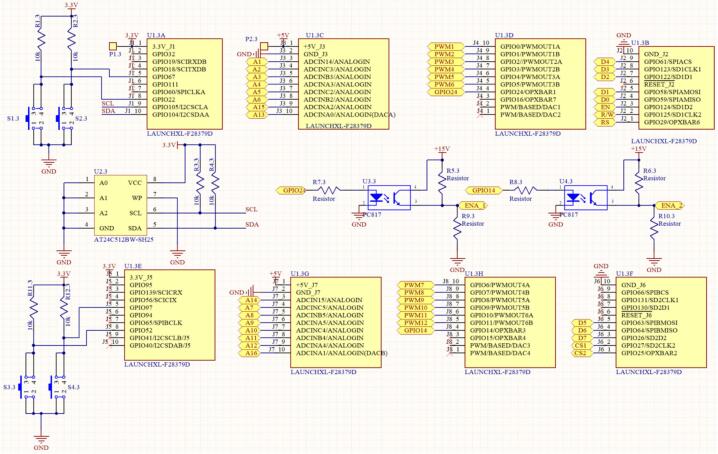
Schematic diagram of the control circuit based on the LAUNCHPAD F28379D.

Fig. 9 illustrates the output pins of the LaunchPad F28379D board in the design. It includes 16 ADC input channels (A1 to A16), 12 PWM output channels (PWM1 to PWM12), and signal pins used for other peripherals.

**Fig. 10:**
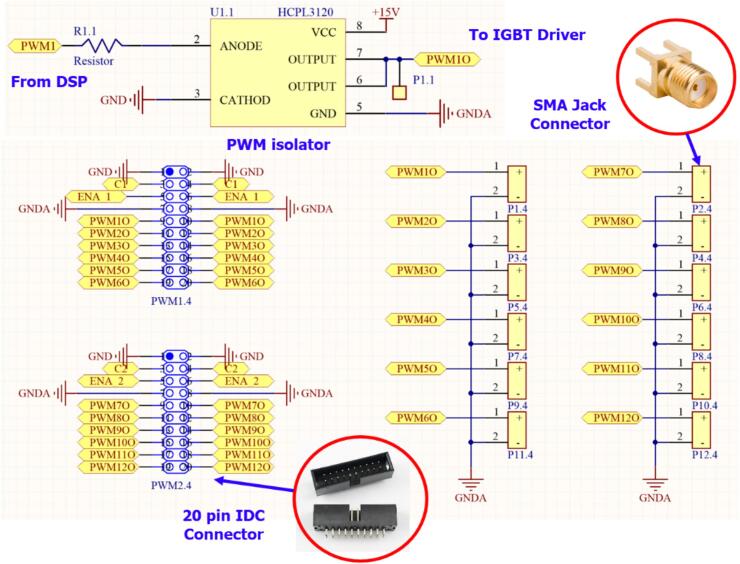
Schematic diagram of the PWM signal connection circuit between the driver board and the control board via a 20-pin IDC connector.

For the PWM output, in the proposed solution, the hardware is designed in a modular format, with connections established through IDC and SMA jack connectors. This increases flexibility in installation, modification, and maintenance of the circuit boards within the energy storage system. Additionally, to minimize electromagnetic interference from the power stage, an optocoupler IC is used to ensure that the control circuit remains unaffected by noise feedback from the power circuit. Fig. 10 presents the schematic diagram of the optically isolated circuit that generates PWM signals for the power circuit using the HCPL3120. The output is connected to the IGBT driver board via a 20-pin IDC connector.

**Fig. 11:**
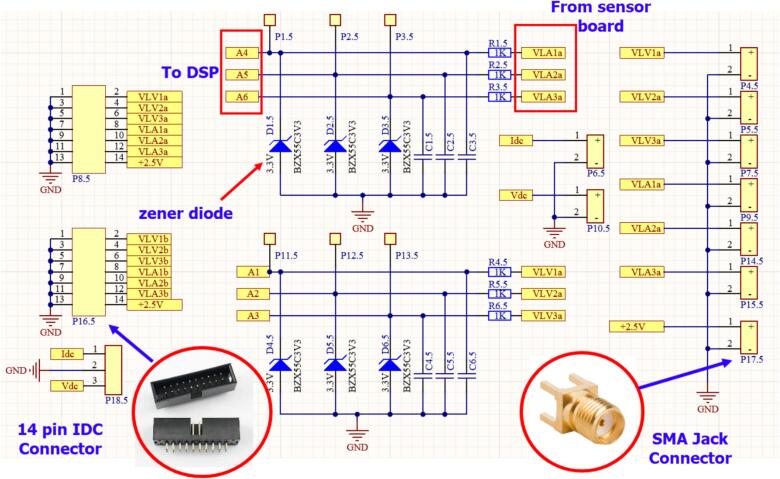
Schematic diagram of the signal connection between the control board and the sensor board.

For the ADC input signals, the control circuit is designed for quick connection to the sensor board via an IDC connector. Additionally, a 3.3(V) Zener diode is placed in parallel with the ADC input of the DSP to protect against signal levels exceeding the measurement range of the DSP F28379D (0–3 V). Furthermore, a pre-designed LC low-pass filter circuit is included to reduce noise in the signal when necessary. Fig. 11 illustrates the schematic diagram of the control circuit with sensor connections through a 14-pin IDC connector.

**Fig. 12:**
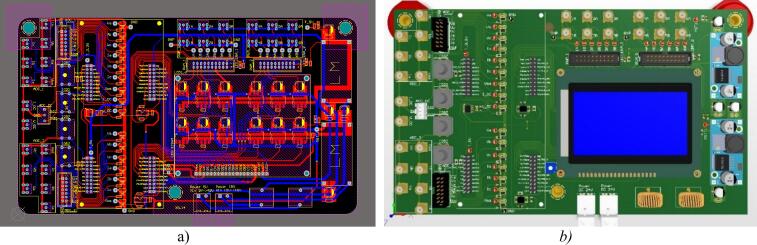
PCB layout and 3D schematic of the proposed control circuit. a) PCB layout. b) 3D model.

The PCB layout design and the 3D model of the control circuit are presented in Fig. 12(a) and (b), respectively.

### 3.5 Isolated voltage & current sensor module

#### 3.5.1 Design and calculation of voltage sensor board

Currently, there are many types of voltage sensors and modules available on the market for measuring voltage values. In research applications, isolated voltage sensors based on the Hall effect are commonly used because they provide high accuracy, good linearity, low error, wide bandwidth, and strong noise immunity. These characteristics make them widely utilized in both laboratory experiments and industrial applications. Table 1 presents the technical specifications of the LV25-P voltage sensor from LEM (Switzerland), which is used in the design.

**Table 1:** Specifications of the LV25-P Voltage Sensor.

Parameter	Symbol	Value	Units
Measuring Range	V_PN_	±10 to ±500	V
Primary nominal RMS current	I_PN_	10	mA
Primary current, measuring range	I_PM_	0…±14	mA
Secondary nominal RMS current	I_SM_	25	mA
Supply Voltage	V_C_	± 12…15	V
Turns ratio	K_N_	2500: 1000	
Isolation RMS Voltage	V_d_	2.5	kV
Bandwidth (−3dB)	BW	DC to 100	kHz
Linearity Error	$\varepsilon_{L}$	<0.2 of Vn	%

**Fig. 13:**
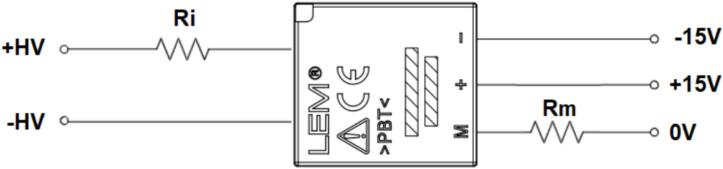
Wiring diagram of the LV 25-P voltage sensor.

In this study, the LV25-P voltage transducer from LEM was selected due to its excellent accuracy, high linearity, wide bandwidth, and strong noise immunity. The design requires a primary RMS current (IPN) of 10(mA), while the primary-side voltage to be measured can range from 10 up to 500(V). The schematic of the LV25-P voltage transducer is presented in Fig. 13.

Where R_i_(Ω) is the primary input resistance and R_m_(Ω) is the secondary output resistance. The measured voltage source is selected as 220(V) at a frequency of 50(Hz). The amplitude of the DC-link voltage is calculated using Equation (5).

(5) $$V_{\mathrm{DCBUS}}=\sqrt{2}∙U_{\mathrm{rms}}=\sqrt{2}∙220=311.126\;\left(V\right)$$

The output waveform of this converter must match the input range of the analog-to-digital converter (ADC) used by the DSP TMS320F28379D. According to Table 1, the voltage conversion ratio of the LV25-P voltage transducer is Np/Ns = 2500:1000. The primary objective is to scale down the input voltage from 220(V) to a level compatible with the 3.3(V) ADC range. Given a primary RMS current of 10(mA), Ohm’s Law is applied using Equation (6).

(6) $$R_{i}=\frac{V_{\mathrm{DCBUS}}}{I_{\mathrm{PN}}}=\frac{311.126}{10mA}=31112.6\;\left(\Omega \right)$$

Since a single 31112.6(Ω) resistor is not readily available, 20 resistors of 2.2(kΩ) each are selected as a replacement. Consequently, the new nominal primary current is calculated using Equation (7). This new nominal current I_PN_ remains within the allowable specification, staying below 10(mA). The power dissipation on each 2.2(kΩ) resistor is determined using Equation (8).

(7) $$I_{\mathrm{PN}}=\frac{V_{\mathrm{DCBUS}}}{\mathrm{Ri}}=\frac{311.126}{44k\Omega }=7.071\;\left(mA\right)$$

(8) $$P_{d}=I_{\mathrm{PN}}^{2}\cdot R={\left(7.071\cdot 10^{-3}\right)}^{2}\cdot 2.2\cdot 10^{3}=0.102\left(W\right)$$

The 2.2(kΩ) series input resistor with a 0.125(W) rating is selected. The design then continues on the secondary side of the voltage transducer. Based on the conversion ratio from the datasheet, Np/Ns = 2500:1000, the secondary RMS current I_SN_ is determined when the primary RMS current IPN is 7.071(mA), as calculated using Equation (9).

(9) $$\frac{N_{p}}{N_{s}}=\frac{I_{\mathrm{SN}}}{I_{\mathrm{PN}}}\;\to I_{\mathrm{SN}}=\frac{N_{P}}{N_{s}}∙I_{\mathrm{PN}}=\frac{2500}{1000}∙7.071=17.677\;\left(mA\right)$$

A resistor Rm = 220(Ω) is selected, which falls within the recommended measurement resistance range specified in the LV25-P datasheet. The output voltage at terminal M is then calculated using Equation (10). Consequently, a 220(Ω) resistor with a 0.125(W) rating is chosen as the measurement resistor Rm on the secondary side. As a result, the 220(V) system voltage is converted to an amplitude value of 3.89(V).

(10) $$V_{M}=I_{\mathrm{SN}}∙R_{m}={17.677∙10}^{-3}∙220=3.89\;\left(V\right)$$

**Fig. 14:**
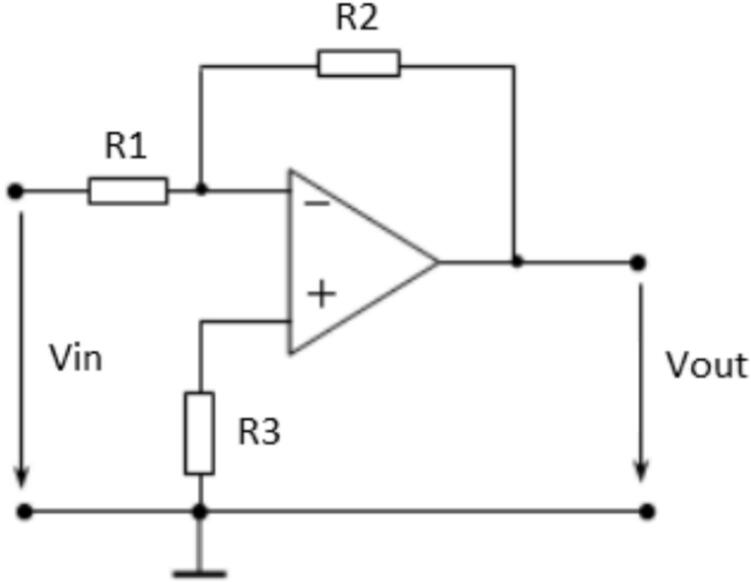
Principle of the inverting amplifier circuit using voltage drift compensation method.

With this output voltage V_M_, OP-AMP circuits are used to scale the signal to a voltage range of 0–3(V), corresponding to the 12-bit DSP ADC range (0–4095). First, an inverting amplifier circuit is used to reduce $V_{M}$, as shown in Fig. 14.

With an input voltage of V_in_ = V_M_ = 3.89(V), the desired output voltage Vout should be less than 1.5(V). First, we select R_2_ = 1(kΩ) and apply the inverting amplifier formula in Equation (11) to calculate the value of R_1_.

(11) $$V_{out}=-\frac{R_{2}}{R_{1}}∙V_{in}\to R_{1}=-\frac{R_{2}}{V_{\mathrm{out}}}∙V_{in}=\frac{1}{1.5}∙3.89=2.59\;\left(k\Omega \right)$$

Selecting R_1_ = 3(kΩ). To minimize the offset voltage drift, the voltage drift compensation method is applied, and R_3_ is determined using Equation (12).

(12) $$R_{3}=\frac{R1∙R2}{R1+R2}=\frac{{3∙10}^{3}∙{1∙10}^{3}}{{3∙10}^{3}+{1∙10}^{3}}=750\;\left(\Omega \right)$$

**Fig. 15:**
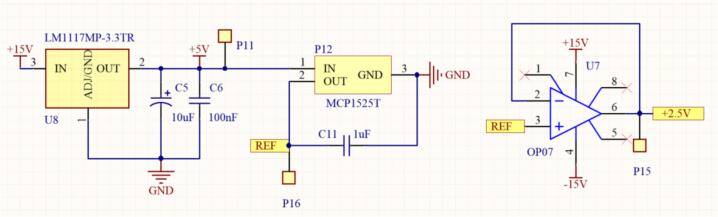
Schematic diagram of the reference voltage generation circuit.

To generate the offset reference voltage, a combination of the AMS1117-5.0 V voltage regulator and the MCP1525T-I/TT REF high-precision reference voltage IC is used, providing an output voltage of Vout = 2.5(V). The schematic diagram of the reference voltage generation circuit for offset is presented in Fig. 15.

**Fig. 16:**
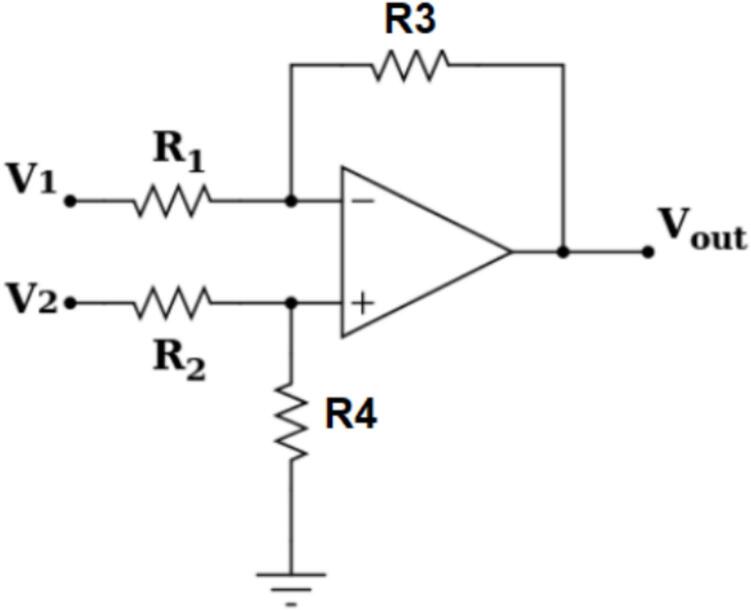
Schematic diagram of the differential amplifier circuit.

To shift the signal to a DC level readable by the DSP F28379D, a differential amplifier circuit is used in this study, as shown in Fig. 16.

To shift the zero point to 1.5(V) from the input voltage V_2_ = 2.5(V), ensuring that the DSP can read the signal when V_1_ is at 0(V), we need V_out_ = 1.5(V) with V_2_ = V_offset_ = 2.5(V). Selecting R_1_ = R_2_ = R_3_ = 1(kΩ), the value of R_4_ is calculated using Equation (13).

(13) $$V_{\mathrm{out}}=V_{2}\left[\frac{\left(R3+R1\right)∙R4}{\left(R4+R2\right)∙R1}\right]-V_{1}∙\left(\frac{R3}{R1}\right)\to 1.5=2.5∙\left[\frac{\left({1∙10}^{3}+{1∙10}^{3}\right)∙R4}{\left(R4+{1∙10}^{3}\right)∙1}\right]-0\to R_{4}=428.571\;\left(\Omega \right)$$

**Fig. 17:**
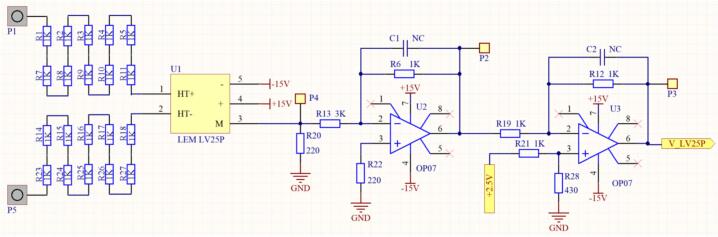
Schematic diagram of the voltage Sensor circuit using the LEM LV25-P.

Selecting R_4_ = 430(Ω). The final schematic diagram of the voltage sensor circuit using the LEM LV25-P is presented in Fig. 17.

#### 3.5.2 Design and calculation of the current sensor board

The LA 25-P current sensor from LEM is used in this study, with its technical specifications presented in Table 2. This sensor, based on the Hall effect, offers high accuracy, excellent linearity, low error, wide bandwidth, and strong noise immunity. It converts high-level AC current signals into low-level current signals that are suitable for reading by the ADC (Analog-to-Digital Converter) of the DSP (Digital Signal Processor) TMS320F28379D.

**Table 2:** Specifications of the LA 25-P current sensor.

Parameter	Symbol	Value	Unit
Nominal Primary Current	I_PN_	25	A
Secondary nominal RMS current	I_SN_	25	mA
Primary current, measuring range	I_PM_	0…± 55	A
Supply Voltage	U_C_	±12…15	V
Conversion Ratio (Np/Ns)	K_N_	1: 1000	
Frequency bandwidth (−1 dB)	BW	DC … 200	kHz
Isolation RMS Voltage	V_d_	3	kV
Linearity error	$\varepsilon_{L}$	< 0.15	%

**Fig. 18:**
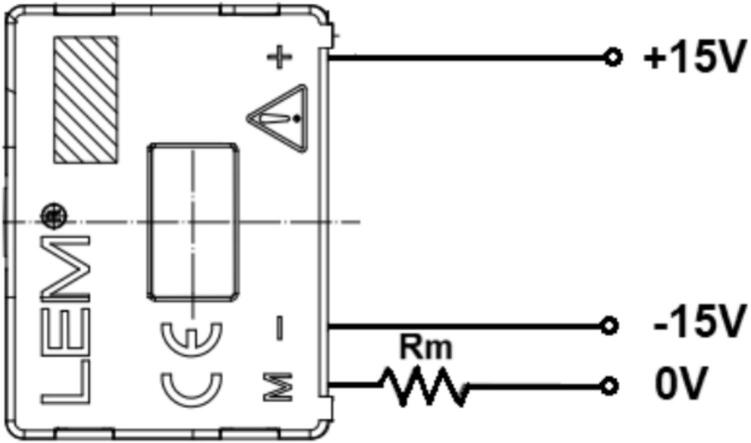
Wiring diagram of the LA 25-P current sensor.

The current sensor design is based on the requirement to convert the input current into a proportional voltage value according to the specified design parameters. The LA 25-P current transducer is implemented in this study due to its advantages, similar to the LV 25-P voltage transducer. According to the LA 25-P specifications, the proposed connection configuration is illustrated in Fig. 18.

Based on this connection configuration, the designed current is 5(A) to enhance the accuracy of current sensing. The LA 25-NP sensor is wound with three turns on the primary side, resulting in an effective primary current of 15(A). With a conversion ratio of 1:1000, the secondary current is calculated using Equation (14).

(14) $$\frac{I_{\mathrm{PN}}}{I_{\mathrm{SN}}}=\frac{1000}{1}\to I_{\mathrm{SN}}=\frac{I_{\mathrm{PN}}}{1000}=\frac{15}{1000}=0.015\left(A\right)$$

The value of the measurement resistor Rm can be determined by applying Ohm’s Law on the secondary side. Given Is = 15(mA), Rm is selected based on the manufacturer’s recommendation, which ranges from 50 to 400(Ω). Choosing R_m_ = 220(Ω), the output voltage is then calculated using Equation (15).

(15) $$V_{m}=I_{\mathrm{SN}}.R_{m}={15∙10}^{-3}∙220=3.3\left(V\right)$$

**Fig. 19:**
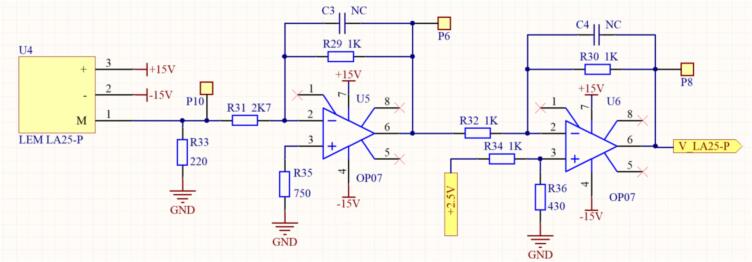
Schematic diagram of the current sensor circuit using the LEM LA25-P sensor.

Therefore, a 220(Ω) resistor with a 0.125(W) power rating is selected as the measurement resistor R_m_ on the secondary side. As a result, the system current of 5(A) is converted into an amplitude value of 3.3(V). The calculations for the amplifier circuit and the offset circuit are similar, and the final design of the current sensor circuit using the LA 25-P sensor is shown in Fig. 19.

**Fig. 20:**
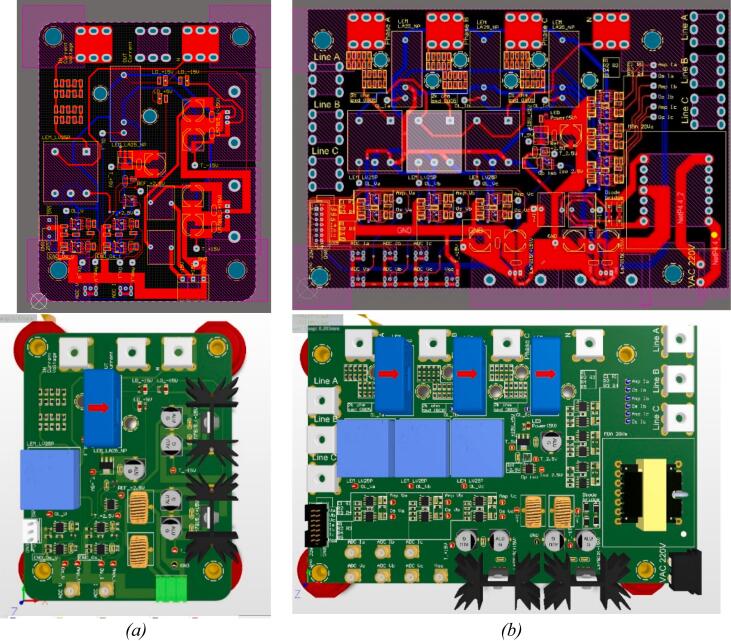
Design results of the current and voltage sensor circuit. a) One phase isolated voltage and current board. b) Three phase isolated voltage and current board.

The design results for the PCB layout and 3D model of the current and voltage sensor boards are shown in Fig. 20. In Fig. 20(a), the sensor circuit designed for a single pair of sensors is used for measuring either single-phase AC circuits or DC circuits. Additionally, Fig. 20(b) shows the sensor circuit with three pairs of sensors for measuring three-phase AC circuits. The sensor outputs are connected to IDC and SMA connectors, making it easy to connect to other circuits or devices, such as easily linking to an oscilloscope.

## 4 Design files summary

The design files for the system of 3-Phase energy storage system using DSP F28379D are shown in Table 3, wherein, the overview descriptions of each are as follows:

• SCH_Hall_1phase_DC: Schematic Diagram of the Current and Voltage Sensor Circuit for Single-Phase.
• PCB_Hall_1phase_DC: PCB Layout Diagram of the Current and Voltage Sensor Circuit for Single-Phase.
• SCH_Hall_3_phase_AC_IDC_SMA_Output: Schematic Diagram of the Output Connection Using IDC and SMA Connectors for the 3-Phase Current and Voltage Sensor Circuit.
• SCH_Hall_3_phase_AC_LA25P: Schematic Diagram of the LA25-P Current Sensor Circuit for 3-Phase Current and Voltage Measurement.
• SCH_Hall_3_phase_AC_LV25P: Schematic Diagram of the LV25-P Voltage Sensor Circuit for 3-Phase Current and Voltage Measurement.
• SCH_Hall_3_phase_AC_Power_Supply: Schematic Diagram of the ± 15 V Regulated Power Supply for the 3-Phase Current and Voltage Sensor Circuit.
• PCB_Hall_3_phase_AC: PCB Layout Diagram of the 3-Phase Current and Voltage Sensor Circuit.
• SCH_F28379D_Controll_Board_ADC: Schematic Diagram of the Sensor Connection in the Control Circuit.
• SCH_F28379D_Controll_Board_DSP: Schematic Diagram of the LaunchPad F28379D Control Circuit Board.
• SCH_F28379D_Controll_Board_ISO_PWM: Schematic Diagram of the PWM Signal Isolation in the Control Circuit.
• SCH_F28379D_Controll_Board_Power_Supply: Schematic Diagram of the Power Supply Section in the Control Circuit.
• SCH_F28379D_Controll_Board_PWM: Schematic Diagram of the IGBT Driver Circuit Connection in the Control Circuit.
• PCB_F28379D_Control_Board: PCB Layout Diagram of the Control Circuit.
• SCH_Power_Board: Schematic Diagram of the Power Board.
• PCB_Power_Board: PCB Layout Diagram of the Power Board.
• Softwave-BESS-charge–discharge-F28379D: The control algorithm program is written in C language and runs in the Code Composer Studio software environment.

**Table 3:** Design files for the system.

Design file name	File type	Open-source license	Location of the file
SCH_Hall_1phase_DC	schematic file, Altium	GNU GPL v3	https://osf.io/mzsn3/files/osfstorage
PCB_Hall_1phase_DC	PCB file, Altium	GNU GPL v3	https://osf.io/mzsn3/files/osfstorage
SCH_Hall_3_phase_AC_IDC_SMA_Output	schematic file, Altium	GNU GPL v3	https://osf.io/mzsn3/files/osfstorage
SCH_Hall_3_phase_AC_LA25P	schematic file, Altium	GNU GPL v3	https://osf.io/mzsn3/files/osfstorage
SCH_Hall_3_phase_AC_LV25P	schematic file, Altium	GNU GPL v3	https://osf.io/mzsn3/files/osfstorage
SCH_Hall_3_phase_AC_Power_Supply	schematic file, Altium	GNU GPL v3	https://osf.io/mzsn3/files/osfstorage
PCB_Hall_3_phase_AC	PCB file, Altium	GNU GPL v3	https://osf.io/mzsn3/files/osfstorage
SCH_F28379D_Control_Board_ADC	schematic file, Altium	GNU GPL v3	https://osf.io/mzsn3/files/osfstorage
SCH_F28379D_Control_Board_DSP	schematic file, Altium	GNU GPL v3	https://osf.io/mzsn3/files/osfstorage
SCH_F28379D_Control_Board_ISO_PWM	schematic file, Altium	GNU GPL v3	https://osf.io/mzsn3/files/osfstorage
SCH_F28379D_Control_Board_Power_Supply	schematic file, Altium	GNU GPL v3	https://osf.io/mzsn3/files/osfstorage
SCH_F28379D_Control_Board_PWM	schematic file, Altium	GNU GPL v3	https://osf.io/mzsn3/files/osfstorage
PCB_F28379D_Control_Board	PCB file, Altium	GNU GPL v3	https://osf.io/mzsn3/files/osfstorage
SCH_Power_Board	schematic file, Altium	GNU GPL v3	https://osf.io/mzsn3/files/osfstorage
PCB_Power_Board	PCB file, Altium	GNU GPL v3	https://osf.io/mzsn3/files/osfstorage
Softwave-BESS-charge–discharge-F28379D	Software file, C Code for DSP	GNU GPL v3	https://osf.io/mzsn3/files/osfstorage

## 5 Bill of materials summary

The costs of components of each board of the system are shown in Table 4, Table 5, Table 6 and Table 7.

**Table 4:** The bill of one-phase isolated voltage and current sensor module.

Designator	Component	Number	Cost per unit (US$)	Source of materials	Material type
C1, C2, C3, C4	CAP CER 0805 22NF 50 V X7R 10 %	4	0.022	Digikey	Electronic
C5	CAP ALUM HYBRID 22UF 20 % 80 V SMD	1	1.29	Digikey	Electronic
C6	CAP CER 0.1UF 35 V X7R 0805	1	0.08	Digikey	Electronic
C7, C8, C12, C13	CAP ALUMHYB 100UF 20 % 50 V SMD	4	1.289	Digikey	Electronic
C9, C10, C14, C15	CAP CER 0.1UF 50 V X7R 0805	4	0.08	Digikey	Electronic
C11	CAP CER 1UF 50 V X7R 0805	1	0.08	Digikey	Electronic
L1, L2	FIXED IND 330UH 3.3A 100 MOHM	2	2.47	Digikey	Electronic
LD1, LD2, LD3	LTST-C191KGKT	3	0.1	Digikey	Electronic
P1, P5	PCB Brass Square Screw Terminals M5	2	0.03	Alibaba	Electronic
P2, P3, P4, P6, P7, P9, P10, P12, P14, P15, P16	PC TEST POINT MINIATURE RED	11	0.337	Digikey	Electronic
P8	Molex CH3.96 3 Pin Male Female Housing Header Connectors	1	0.2	Aliexpress	Electronic
P11	MCP1541T	1	1.01	Digikey	Electronic
P13	Pitch 5.08 mm 3P Screw Plug-in PCB Terminal Block	1	0.12	Aliexpress	Electronic
R1, R2, R3, R4, R5, R6, R7, R8, … R31, R32, R33, R34, R35, R36	2.2kΩ Chip Resistor − Surface Mount 0805	36	0.007	Digikey	Electronic
R37, R38, R39	RES 330 OHM 1 % 1/4W 1206	3	0.02	Digikey	Electronic
SW1	3 Position Header Connector (2.54 mm) Through Hole	1	0.33	Digikey	Electronic
U1	LEM LV 25-P	1	27.3	Ebay	Electronic
U2, U3, U5, U6, U7	OP07DDR	5	1.12	Digikey	Electronic
U4	LEM LA 25-P	1	21.7	Ebay	Electronic
U8	LM1117MP-3.3	1	1.38	Digikey	Electronic
U9	LM7815CT	1	1.74	Digikey	Electronic
U10	LM7915CT	1	1.48	Digikey	Electronic
**Total cost: 71.2 ($)**

**Table 5:** The bill of three-phase isolated voltage and current sensor module.

Designator	Component	Number	Cost per unit (US$)	Source of materials	Material type
C1, C2, C3, C4, C5, C6, C7, C8, C9, C10, C11, C12	CAP CER 0805 22NF 50 V X7R 10 %	12	0.022	Digikey	Electronic
C13, C14, C17, C18, C21	CAP ALUMHYB 100UF 20 % 50 V SMD	5	1.289	Digikey	Electronic
C15, C16, C19, C20, C22, C23	CAP CER 0.1UF 35 V X7R 0805	6	0.08	Digikey	Electronic
L1, L2	FIXED IND 330UH 3.3A 100 MOHM	2	2.47	Digikey	Electronic
LD1, LD2, LD3	LTST-C191KGKT	3	0.1	Digikey	Electronic
P1, P3, P4, P5, P6, P7, P8	RF Connectors / Coaxial Connectors SMA Female PCB Edge Mount	8	1.8	Mouser	Electronic
P2	Headers & Wire Housings SEK18 SVML STD STR29 RLG 14P AU0.76	1	2.19	Mouser	Electronic
P9, P10, P13, P14, P15, P18, P19, P20, P22	PC TEST POINT MINIATURE RED	9	0.337	Digikey	Electronic
P24, P25, P27, P31, P32, P33, P34, P35, P36, P37, P38, P41, P42, P44, P45, P46	PC TEST POINT MINIATURE BLUE	16	0.337	Digikey	Electronic
P40	Pitch 5.08 mm 3P Screw Plug-in PCB Terminal Block	1	0.12	Aliexpress	Electronic
P43	MCP1541T	1	1.01	Digikey	Electronic
R1, R2, R3, R4, R5, R6, R7, R8, R9, …. R109, R110, R111	2.2kΩ Chip Resistor − Surface Mount 0805	127	0.007	Digikey	Electronic
U1, U4, U7	LEM LA 25-P	3	21.7	Ebay	Electronic
U2, U3, U5, U6, U8, U9, U11, U12, U14, U15, U17, U18, U22	OP07DDR	13	1.12	Digikey	Electronic
U10, U13, U16	LEM LV 25-P	3	27.3	Ebay	Electronic
U19	LM7815CT	1	1.74	Digikey	Electronic
U20	LM7915CT	1	1.48	Digikey	Electronic
U21	LM1117MP-3.3	1	1.38	Digikey	Electronic
**Total cost: 205.6 ($)**

**Table 6:** The bill of control board based on F28379D LaunchPad.

Designator	Component	Number	Cost per unit (US$)	Source of materials	Material type
C1, C2, C3, C4, C5, C6, C7, C8, C9, C10, C11, C12, C13, C14, C15, C16	CAP CER 0805 22NF 50 V X7R 10 %	16	0.022	Digikey	Electronic
C17, C22, C18, C23	CAP ALUMHYB 100UF 20 % 50 V SMD	4	1.289	Digikey	Electronic
C19, C20, C21, C24	CAP CER 0.1UF 50 V X7R 0805	4	0.08	Digikey	Electronic
C25, C26, C27, C28, C29, C30, C31, C32, C33, C34, C35, C36	CAP ALUMHYB 47UF 20 % 50 V SMD	12	1.12	Digikey	Electronic
D1, D2, D3, D4, D5, D6, D7, D8, D9, D10, D11, D12, D13, D14, D15, D16	DIODE ZENER 3.3 V 375 MW SOD123F	16	0.1	Digikey	Electronic
IC1, IC2	LM2596S DC-DC ADJUSTABLE STEP-	2	7.95	Digikey	Electronic
L1, L2	FIXED IND 330UH 3.3A 100 MOHM	2	2.47	Digikey	Electronic
LCD1	Graphic LCD Display Module Transmissive STN − Super-Twisted Nematic Parallel, 8-Bit 128 x 64	1	27.84	Digikey	Electronic
LD1, LD2, LD3, LD4	LTST-C191KGKT	4	0.1	Digikey	Electronic
P1, P2, P4, P11, P13, P14, P22, P23, P24, P25, P27, P28, P32, P33, P34, P35, P36, P37, P38, P39, P40, P41, P42, P43, P44, P45, P46, P47, P48, P49, P50, P54	PC TEST POINT MINIATURE RED	32	0.337	Digikey	Electronic
P3, P5, P6, P7, P8, P10, P12, P15, P17, P18, P20, P21, P26, P29, P30, P31, P58, P59, P60, P61, P62, P63, P64, P65, P66, P67, P68, P69	RF Connectors / Coaxial Connectors SMA Female PCB Edge Mount	27	1.8	Mouser	Electronic
P9, P16	Headers & Wire Housings SEK18 SVML STD STR29 RLG 14P AU0.76	1	2.19	Mouser	Electronic
P19	Pitch 5.08 mm 3P Screw Plug-in PCB Terminal Block	1	0.12	Aliexpress	Electronic
P51, P55	Pitch 5.08 mm 2P Screw Plug-in PCB Terminal Block	1	0.12	Digikey	Electronic
P52, P53, P56, P57	PC TEST POINT MINIATURE BLUE	4	0.337	Digikey	Electronic
PWM1, PWM2	Connector Header Through Hole 20 position (1.27 mm)	2	0.875	Digikey	Electronic
R1, R2, R3, R4, R5, R6, R7, R8, … R42, R43, R44, R45	1kΩ Chip Resistor − Surface Mount 0805	38	0.007	Digikey	Electronic
R17, R18, R19, R20, R27, R28	10kΩ Chip Resistor − Surface Mount 0805	6	0.007	Digikey	Electronic
S1, S2, S3, S4	MULTICOLOR BUTTONS − 4-PACK	4	0.45	Digikey	Electronic
U1	LAUNCHPAD TMS320F2837XD/2837XS	1	46.8	Digikey	Electronic
U2	AT24C512BW-SH25 IC EEPROM 512KBIT I2C 1MHZ 8SOIC	1	1.24	Digikey	Electronic
U3, U4	PC817X2NSZ9F Optoisolator Transistor Output 5000Vrms 1 Channel 4-DIP	2	0.27	Digikey	Electronic
U5, U6, U7, U8, U9, U10, U11, U12, U13, U14, U15, U16	HCPL-3120-500E OPTOISO 3.75KV 1CH GT DVR 8DIP	12	3.41	Digikey	Electronic
VR1	3362P-1-103LF TRIMMER 10 K OHM 0.5 W PC PIN TOP	1	0.91	Digikey	Electronic
**Total cost: 227.4 ($)**

**Table 7:** The bill of power board.

Designator	Component	Number	Cost per unit (US$)	Source of materials	Material type
C1, C2, C3, C4	CAP ALUM 330UF 20 % 450 V SNAP TH	4	11.69	Digikey	Electronic
C5, C6, C7, C8	CAP FILM 1UF 10 % 400VDC RADIAL	4	1.26	Digikey	Electronic
C9, C10, C11	CAP FILM 2.2UF 10 % 400VDC RADIAL	3	1.07	Digikey	Electronic
C12, C13, C14, C15, C16, C17	CAP CER 1812 10NF 1000 V X7R 10 %	6	0.74	Digikey	Electronic
Header_M1, Header_M2, Header_M3, Header_M7, Header_M10, Header_M11, Header_M12, Header_M13, Header_M, T1_IN, T1_OUT, T2_OUT, T3_OUT	Terminals SCREW TERMINAL	13	0.6	Mouser	Electronic
J1, J2, J3, J4, J5, J6	Headers & Wire Housings 0.156 SPOX HEADER	1	0.5	Mouser	Electronic
Q1, Q2, Q3, Q4, Q5, Q6	IGBTs 1200 V 40A FS2 Trench IGBT	6	9.07	Mouser	Electronic
R1, R2, R3, R4, R5, R6	LTST-C191KGKT	6	0.26	Mouser	Electronic
**Total cost: 123.7 ($)**					

## 6 Build instructions

**Fig. 21:**
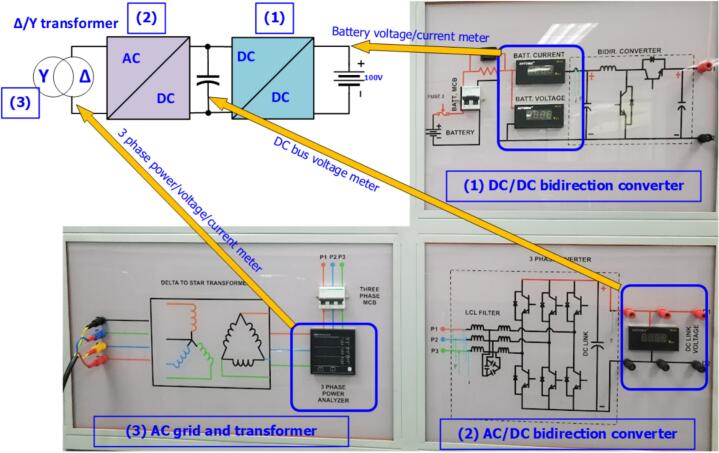
Front view of the experimental model for the energy storage system.

In this study, a complete experimental model is used to visualize the hardware configurations, as well as integrate protection switches and measuring instruments for experimental calculations in laboratory exercises. Fig. 21 shows the front view of the experimental model, which includes the bidirectional DC/DC converter module, the bidirectional AC/DC converter, the transformer, and the electrical meters used for system measurements in the experiment.

**Fig. 22:**
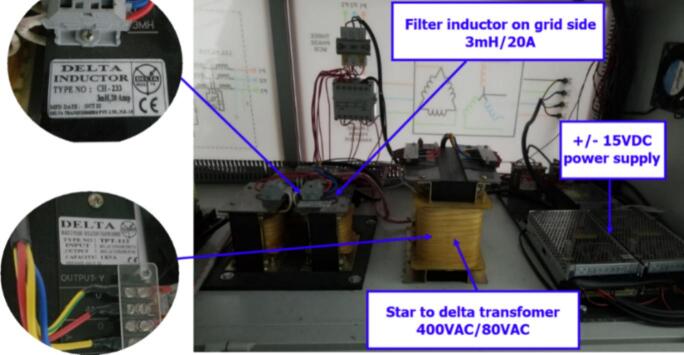
Rear view of the experimental model.

The rear view of the experimental model shows the installation of a Δ/Y transformer to step down the voltage from 80 to 400(V), ensuring voltage reduction at the grid connection point and electrical safety for the wiring setup. The output filter coil used for grid synchronization employs an L-filter with an inductance of 3(mH) and a maximum current of 20(A). A ± 15(V) power supply is used to power the sensor board and the IGBT driver circuit. Fig. 22 illustrates the equipment installed on the rear side of the experimental model.

**Fig. 23:**
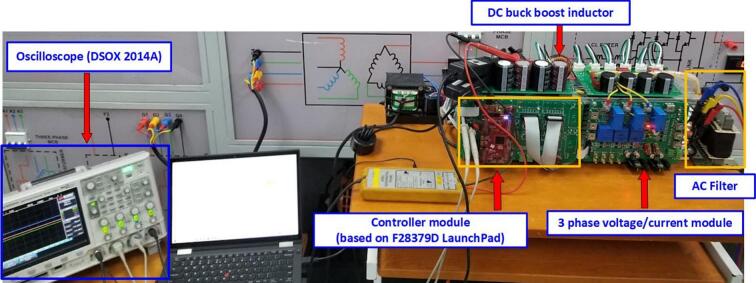
The hardware model mounted heatsink.

The control and measurement modules are designed with appropriate dimensions to be mounted on the SEMIPACK heatsink. Fig. 23 shows the assembly diagram of the sensor circuit and the control circuit into the model. The output of the measurement circuit can be connected to the DSOX 2014A oscilloscope from Keysight through test points or pre-soldered SMA connectors.

**Fig. 24:**
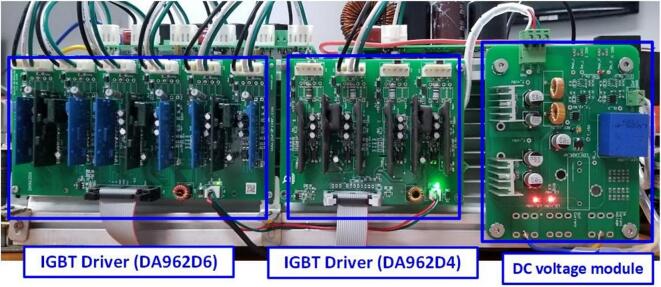
Wiring diagram of power board, DC sensor board and IGBT driver board.

The IGBT driver circuits are purchased from the supplier eBay.com, including the 4-channel PWM driver (DA962D4) for controlling the IGBT in the DC/DC converter and the 6-channel PWM driver (DA962D6) for controlling the IGBT in the DC/AC inverter. Fig. 24 shows the installation of the IGBT gate driver into the heatsink and its connection to the power board. The IGBT driver circuit DA962 features a 20-pin IDC connector input that can be directly plugged into the DSP controller board. Some advantages of this IGBT driver circuit include overcurrent protection through monitoring of the collector-emitter voltage (V_CE_) of the IGBT, electrical isolation between the power and control circuits, a low-voltage gate drive V_CE_ = −7(V) to minimize the influence of parasitic gate capacitance, and configurable input voltage levels with short-circuit current protection via the integrated DESAT protection circuit.

## 7 Operation instructions

**Fig. 25:**
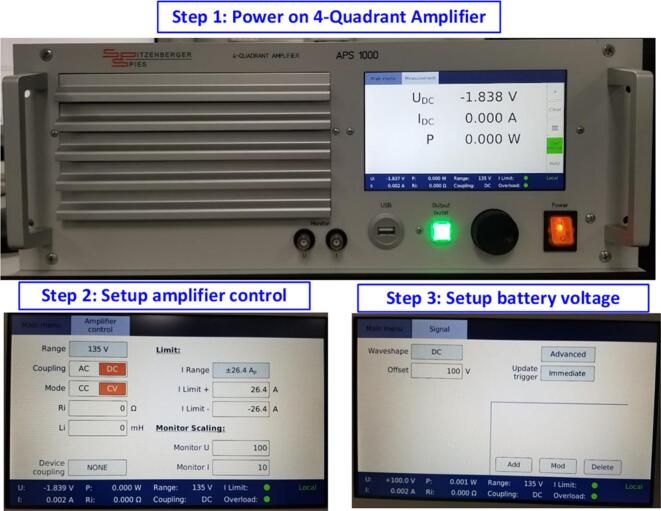
Steps to set up the simulated battery parameters using the APS1000 power supply.

First, it is necessary to connect and power the energy storage battery for the energy storage system. To ensure safety and protect against overcurrent and short-circuit faults, the experimental model of the energy storage system uses a 4-quadrant amplifier power supply as a substitute for the battery. The maximum current is set to ± 26(A), and the nominal voltage of the battery is set to 100(V). Fig. 25 illustrates the steps to set up the simulated battery system using the APS1000 power supply.

Next, the control circuit is powered by connecting the AC/DC power supply plugs, which step down the voltage from 220 V AC to 5 V and ±15 V. After powering the system, the programming of the control board is carried out using the CCS (Code Composer Studio) software from Texas Instruments. When the program is running, if the enable/disable PWM button is not pressed, the power circuit will not be switched on or off.

**Fig. 26:**
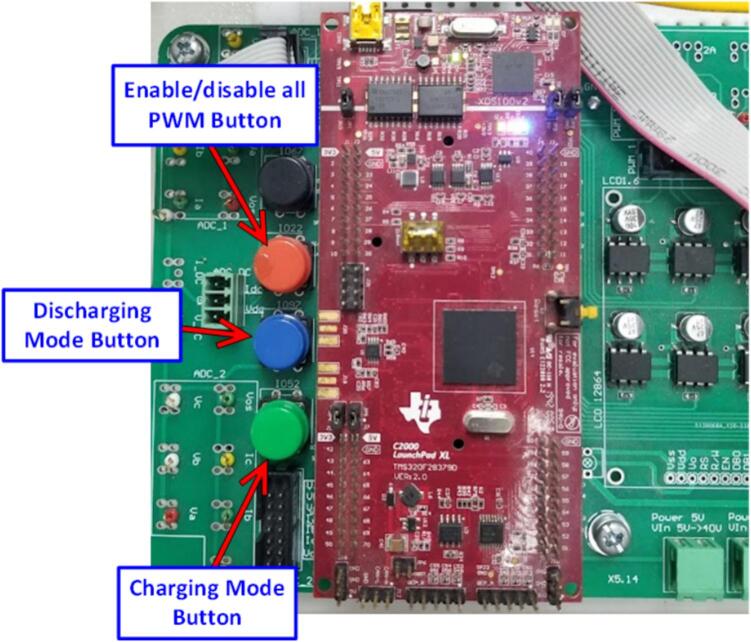
Control of the Energy Storage System operating mode via the push button.

Fig. 26 illustrates the positions of the control buttons for operating modes on the control circuit. The program is pre-programmed to control the power functions of the storage system in two modes as follows:

• Discharging Mode: This mode is activated by pressing the discharging mode button. In this mode, the controller manages the DC/DC boost converter to stabilize the DC bus voltage at 150(V) and performs DC/AC inversion for grid synchronization with a set active power output of 230(W).
• Charging Mode: This mode is activated by pressing the charging mode button. In this mode, the controller manages the bidirectional DC/AC converter in rectifier mode to stabilize the DC bus voltage at 150(V) and controls the DC/DC buck converter to consume 230(W) of active power from the grid.

**Fig. 27:**
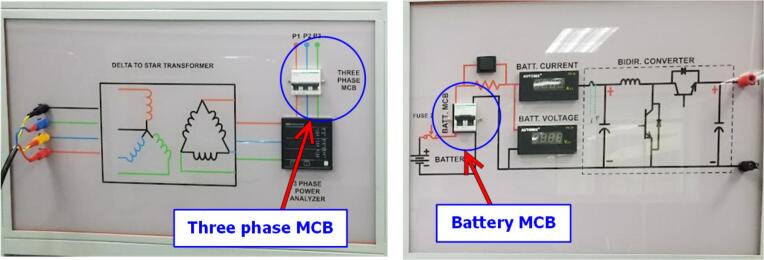
Connect the 3-phase grid and the battery to the power board.

To perform the control circuit operation tests, 3-phase AC power is supplied to the inverter by closing the 3-phase circuit breaker (CB). Similarly, the storage battery is connected to the DC/DC converter by closing the 1-phase circuit breaker (CB), as shown in Fig. 27.

Finally, press the enable/disable PWM button to allow the PWM signal to be sent to the power circuit. To operate the system in charging mode, press the charging mode button, and to operate in discharging mode, press the discharging mode button. When stopping the control, press the enable/disable PWM button, and the PWM signals will be completely disabled.

## 8 Validation and characterization

### 8.1 Sensor board evaluation and testing

**Fig. 28:**
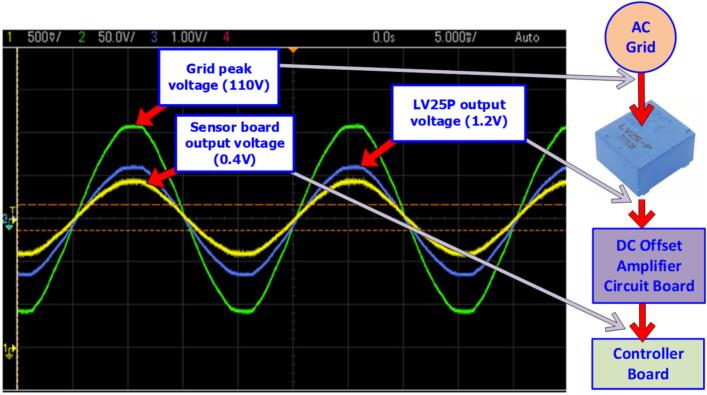
Voltage signal waveform measured in the voltage sensor circuit.

The sensor circuit’s linearity is evaluated by measuring the input and output waveforms. Fig. 28 shows the input waveform of the voltage sensor at a grid voltage of 110(V). The signal passing through the LA25P sensor has a peak value reduced to 1.2(V). The signal then continues into the offset amplifier circuit, with an offset voltage of 1.5(V), and the peak value reaches 0.4(V). Therefore, the gain of the sensor circuit is approximately K_V_ = 275.

**Fig. 29:**
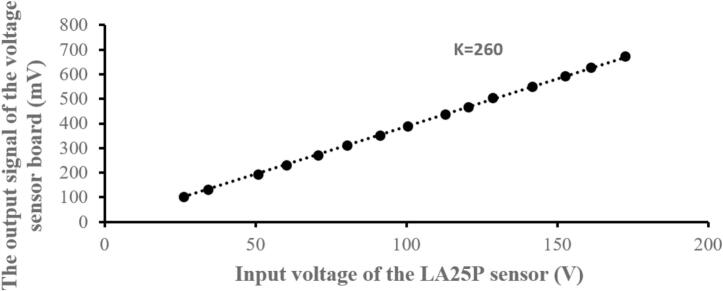
The linearity measurement results for the voltage sensor circuit.

**Fig. 30:**
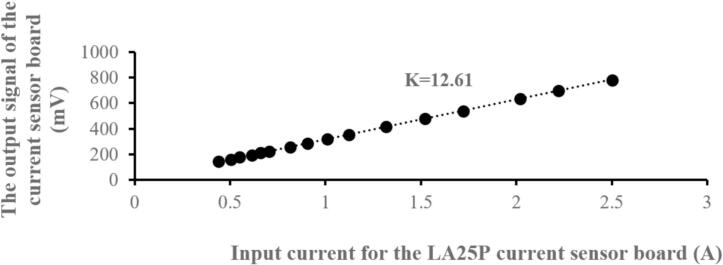
The linearity measurement results for the current sensor circuit.

The sensor circuit experiment was performed for voltages ranging from 0 to 180(V) and currents from 0.5 to 2.5(A) to determine the gain factor and linearity for calibration of the measurement in the control circuit. The measurement results are shown in Fig. 29 and Fig. 30, which correspond to the linearity of voltage and current, respectively. The measured voltage gain was K_V_ = 260, and the measured current gain was K_I_ = 12.61. The results demonstrate that this sensor circuit has good linearity.

In summary, the sensor voltage and current board demonstrated excellent linearity, meaning the output signal remained consistent with changes in the input current across the entire tested range.

### 8.2 Experimental results of the energy storage system

The experimental results of the energy storage system typically focus on how well the system performs under different charging and discharging conditions, including stability, efficiency, and response time. In the experiment, the system parameters of the energy storage system are shown in Table 8. The test AC grid voltage is selected at different levels to simulate grid voltage sag. The control power is set to a fixed value of 230(W) for both the charging and discharging processes.

**Table 8:** Experimental parameters of the energy storage system used for practical research.

Item	Symbol	Value
Simulated battery voltage	V_BAT_	100(V)
Point of Common Coupling voltage in grid connection	V_GRID_	50(V)
DC bus voltage	V_DC_	150(V)
Buck/boost inductor	L_DC_	1.5(mH)
Output inverter filter	L_AC_	3.5(mH)
DC link capacitor (4*330uF)	C_DC_	1320(uF)
Switching frequency	f_sw_	20(kHz)

*Experimental charging test:*		
Controlled charging power	P_charge_	230(W)
Grid connection PCC voltage	V_GRID_	45.8(V)

*Experimental discharging test:*		
Controlled discharging power	P_discharge_	230(W)
Grid connection PCC voltage	V_GRID_	51(V)

**Fig. 31:**
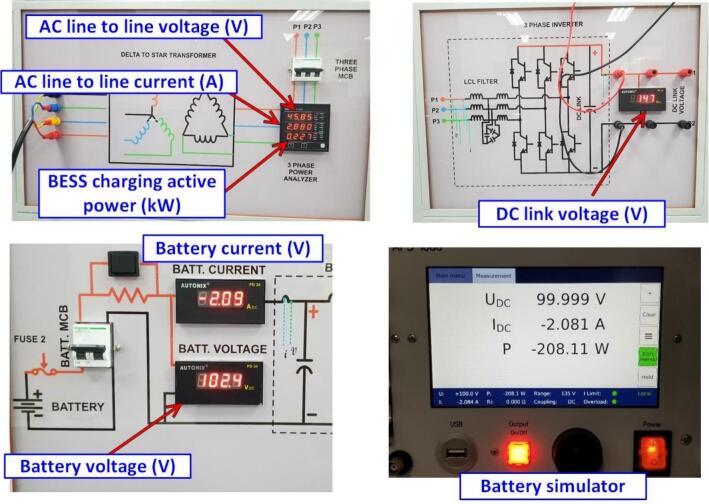
Experimental results of the Energy Storage System Operating in charging mode.

The charging test was conducted at an AC charging power of 230(W) with a grid voltage of 45.8(VAC). The measurement results from the charging test, according to the experimental parameters, are shown in Fig. 31. The DC current and voltage on the battery and battery pack were measured using the PD34 meter from Autonix. Additionally, the power, current, and voltage of the grid were analyzed and measured using the RISH Master 3430 m. The results show that the battery charging power is −208.1(W), with a stable charging power of 227(W). The system efficiency is approximately 91.6 %, and the operating DC bus voltage is 147(V).

**Fig. 32:**
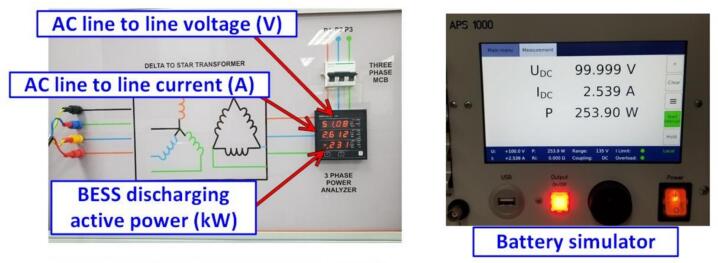
Experimental results of the Energy Storage System Operating in discharging mode.

The measurement results from the discharging test, according to the experimental parameters, are shown in Fig. 32. The discharging test was conducted at a controlled AC discharging power of 230(W) with a grid voltage of 51(V). The results show that the actual discharging power was −231(W). The battery discharging power reached 253.9(W), with a discharging process efficiency of 90.9(%).

**Fig. 33:**
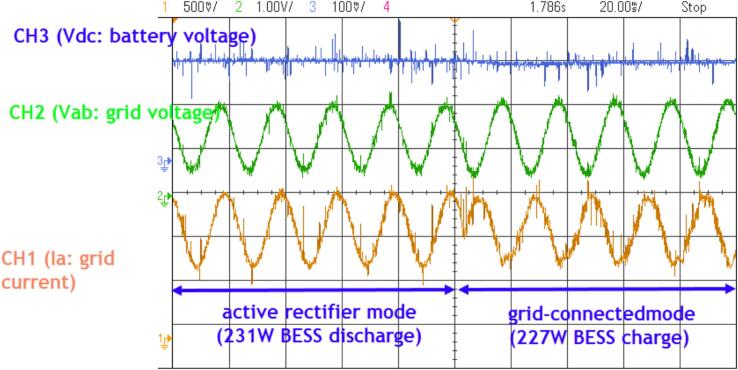
Waveform of the DC bus voltage, current, and grid phase voltage during the control transition from discharging to charging.

The waveform of the DC bus voltage (Vdc), grid line voltage (Vab), and current measured from the grid as observed on the oscilloscope is shown in Fig. 33. The results indicate that the control algorithm from discharging to charging transitions smoothly and stabilizes well within one cycle, with an overshoot close to zero. The voltage across the battery shows minimal change within a 40(ms) period during the transition from the discharging process to the charging process.

**Fig. 34:**
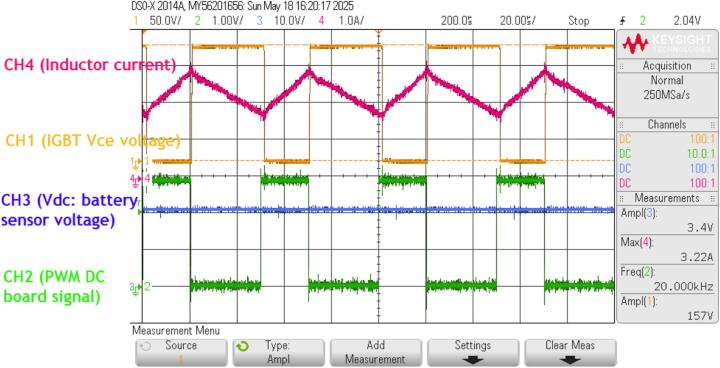
Waveform on the DC/DC power conversion circuit.

Fig. 34 presents the test results of the DC/DC circuit operating in boost mode, with an input battery voltage of 100(V) and an output voltage of 150(V). The switching frequency from DSP F28379 is set at 20(kHz), directly measured at the DSP output (CH2 channel). The waveform at the CE terminal of the IGBT is observed using an isolated probe AP031 Lecroy (CH1 channel). The results show that the switching voltage spike on the IGBT is less than 5(%), and the current ripple through the inductor (CH4 channel) reaches 35(%) with the selected 1.5(mH) boost inductor.

**Fig. 35:**
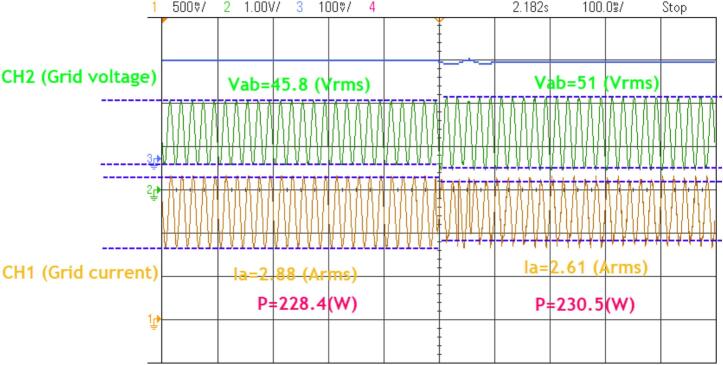
The waveform of the grid current and voltage when the voltage increases by 10%.

The experiment, conducted with a 10 % variation in the grid voltage (from 45.8 Vrms to 50.1 Vrms), shows that the power adjustment has an error margin of approximately 3 %. Fig. 35 presents the waveform result of the 230(W) power control over 50 cycles when the voltage changes.

**Fig. 36:**
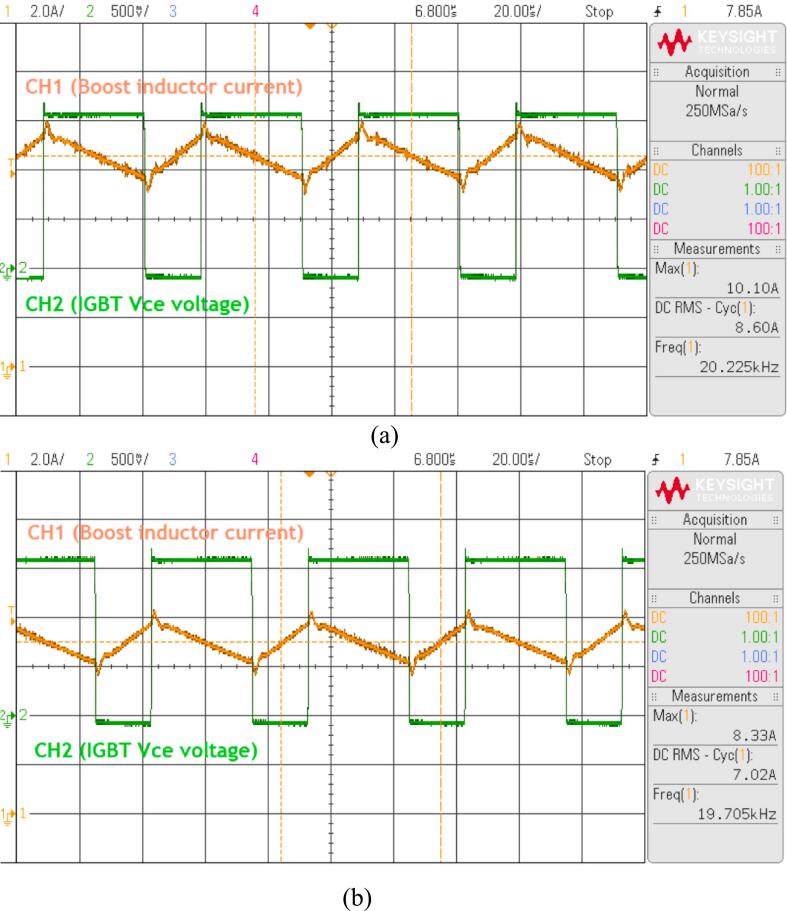
Overload testing on the simulated battery using the APS1000 4-quadrant amplifier power supply. a) With a peak current of 8.33(A). b) With a peak current of 10.1(A).

In this study, the use of the APS1000 4-quadrant amplifier power supply to simulate the energy storage battery is limited in terms of power capacity, as the maximum power of this power supply is 1000(W). The experiment with the battery discharge current through the coil at 7.02(A) at 100(V), with a peak current of 8.33(A) and the current waveform shown in Fig. 36a, will overload the power supply within 2–3 min. Similarly, the experiment with the battery discharge current through the coil at 8.06(A) at 100(V), with a peak current of 10.1(A) and the current waveform shown in Fig. 36b, will overload the power supply within 3 to 5 s. Therefore, we recommend that experiments following this proposed model should be conducted at a power level below 500(W).

In conclusion, the paper presents the design and development of an energy storage system. To support research and experimental practice, the system is designed in a modular form, offering advantages such as easy installation, maintenance, and flexibility in adjusting the power circuit configuration. Specifically, the modules are neatly and simply mounted on a SEMIPACK industrial-standard heat sink from Semikron. Additionally, the modules are conveniently interconnected using IDC connectors and SMA jacks. The system’s operational parameters are measured with industrial meters and visually displayed on a clear acrylic board, facilitating data collection for research purposes. Safety is carefully considered in the study, and the system is specifically designed to operate at a low voltage of under 100(V) through an isolation transformer. This design minimizes the risk of electric shock and fire hazards, ensuring safer wiring and testing of various control programs under different operating conditions. Similarly, both single-phase and three-phase sensor circuits incorporate isolation using Hall current/voltage sensors. The IGBT driver circuit is selected for its ability to provide low-level voltage triggering at −7(V), which reduces noise interference from the parasitic capacitance of the IGBT. Additionally, it offers overcurrent protection by monitoring the collector-emitter voltage (VCE), triggering a fault to shut down the device if necessary. The control circuit is based on the LaunchPad F28379D board, which provides powerful computational capabilities to support the development of complex control algorithms for research. Experimental results demonstrate that the energy storage system operates efficiently, maintaining stable power control within approximately 3 % during both charging and discharging states, even under grid voltage variations of about 10 %. The overall system efficiency achieved is 91.6 % in the charging state and 90.9 % in the discharging state.

## CRediT authorship contribution statement

**Hoai Phong Nguyen:** Writing – review & editing, Writing – original draft, Software, Methodology, Conceptualization. **Thuan Thanh Nguyen:** Writing – review & editing, Writing – original draft, Project administration, Methodology. **Minh Phuong Le:** Writing – review & editing, Supervision, Methodology. **Minh Tan Tran:** Writing – review & editing. **Cong Duy Pham:** Writing – review & editing.

## Declaration of competing interest

The authors declare that they have no known competing financial interests or personal relationships that could have appeared to influence the work reported in this paper.
